# Construction of a novel prognostic model in skin cutaneous melanoma based on chemokines-related gene signature

**DOI:** 10.1038/s41598-023-44598-2

**Published:** 2023-10-24

**Authors:** Xiaoxia Ding, Wenwen Wang, Xiaohua Tao, Zhiming Li, Youming Huang

**Affiliations:** 1 Center for Plastic and Reconstructive Surgery, Department of Dermatology, Zhejiang Provincial People’s Hospital, Affiliated People’s Hospital, Hangzhou Medical College, Hangzhou, Zhejiang China; 2 Department of Dermatology and Venereology, The First Affiliated Hospital of Wenzhou Medical University, Wenzhou, 325000 Zhejiang China

**Keywords:** Cancer, Computational biology and bioinformatics, Immunology, Diseases

## Abstract

Skin cutaneous melanoma, SKCM, is one of the most aggressive treatment-resistant tumours. Despite the fact that the BRAF oncogene and immunological checkpoints such as PD-1/PD-L1 and CTLA-4 have enhanced the therapeutic efficacy of SKCM, the subsequent resistance mechanisms and remedies have raised concerns. Chemokines have a significant role in the immunological milieu of tumor, which may increase the efficacy of checkpoint blockade and serve as a possible therapeutic intervention route. However, there is still no chemokine-based typing and risk model to provide a prognosis and therapeutic efficacy assessment for SKCM patients. In this study, we verified the distinct differences of prognostic stratification as well as immune characteristics between two chemokine-related clusters in SKCM patients. Two clusters of DEGs were discovered to be primarily enriched in B and T cell receptor signaling pathways as well as TNF signaling via NF-kappa-B. Based on 14 prognosis-related DEGs from aforementioned two clusters (*CCL8*, *GBP2*, *GBP4*, *SRNG*, *HLA-DMB*, *RARRES3*, *HLA-DQA1*, *PARP12*, *APOL3*, *IRF1*, *HLA-DRA*, *UBE2L6*, *IL2RA* and *CD38*), a chemokine-related 14-gene prognostic model was established. At the same time, researchers explored differences between the low-risk and high-risk groups in clinical traits, the proportion of infiltration of 22 different types of immune cells, and how well medications worked. The risk score model’s immunotherapy and prognostic predictions were also confirmed in testing groups. Based on the finding, we can claim that there is a clear link between chemokines and TME in SKCM. The risk score may perform as a trustworthy prediction model, giving therapeutic benefits for both chemotherapy and immunotherapy, as well as being beneficial for clinical decision making in SKCM patients.

## Introduction

Over the past ten years, the incidence of skin cutaneous melanoma (SKCM), the most malignant skin tumor, has increased^1^. Melanoma is often diagnosed by clinical examination and histopathology, followed by surgery, radiotherapy or targeted therapy depending on the stage of the disease^2,3^. Despite significant advances in melanoma treatment brought by targeted therapies such as BRAF inhibitors, MEK inhibitors, and immune checkpoint inhibitors (ICIs) including programmed cell death protein 1 (PD-1)/programmed death ligand 1 (PD-L1) and cytotoxic T-lymphocyte-associated protein 4 (CTLA-4), the 5-year survival rate remains around 60%^4,5^. Furthermore, most patients relapse during treatment as a result of medication resistance mechanisms that have evolved^6,7^. Understanding the potential primary routes for manipulating the correlated processes has significant therapeutic implications, perhaps contributing to the development of treatments with fewer side effects and higher clinical effectiveness.

The tumor microenvironment (TME) is essential in the anti-tumor response, and chemokines may modify the TME in tumor^8^. Actually, not only the chemokines in TME, circulating chemokines can also alter tumor cell proliferation, invasion and metastasis^9–11^. Chemokines are a wide class of chemotactic-released cytokines whose primary purpose is to mediate leukocyte directed migration. Both tumor and stromal cells express cognate receptors. Their altered expression controls angiogenesis, cancer cell proliferation, metastasis, and leukocyte activation at all disease stages^12–14^. The chemokine system has become a promising immunotherapy target in recent years^15,16^. The chemokine network provides a possible therapeutic avenue for both improving anti-tumor immunity by encouraging effector cell recruitment as well as penetration of tumor tissue and preventing pro-tumor activities by limiting the recruitment of immunosuppressive cells. Numerous preclinical studies have been carried out in order to boost the susceptibility of efficient anti-tumor immune cells to ICB therapy by modulating chemokines and their receptors, such as CD8+ T cells^17–20^.

The five main subfamilies of chemokines are CXC, CC, CX3C, XC, and CX^21^. Several earlier studies have discovered chemokines to be associated with the development of SKCM. For instance, it was discovered that there was a positive link between immune cell infiltration and CXC subfamily expression in SKCM^22^. Few studies have, however, looked at the possible benefits of chemokine therapy guidance in SKCM. Consequently, analysis of the antitumor immunological response and prognostic function of chemokines in SKCM is required. Further, developing a prognostic model to anticipate treatment proficiency in SKCM is also significant and could give advantages to coordinating clinical applications.

In this study, we attempt to identify distinct chemokine-related clusters in the integrated SKCM dataset from TCGA as well as the GEO database. Subsequently, the differences between the two aforementioned subsets were analyzed, including the molecular characteristics, prognosis, and intensity of immune cell infiltration. Furthermore, prognostic DEGs between the aforementioned clusters were obtained, and based on these DEGs, a chemokine-related risk score model was constructed. After that, the model's ability to forecast clinical outcome and immunotherapeutic effect was examined, both in the training and test cohorts. Based on all these efforts, we hope that this study will benefit the further development of more effective treatments for SKCM patients.

## Materials and methods

### Data collection and processing

470 TCGA-SKCM samples and profiles of gene expression statistics, clinical data, and mutation details were obtained using UCSC-XENA (https://xenabrowser.net/datapages/). Similar to this, the GSE54467 (GPL6884, n = 79) dataset's 79 SKCM samples, clinical information, and gene expression statistics were retrieved from the GEO database (https://www.ncbi.nlm.nih.gov/geo/query/acc.cgi?acc=GSE54467). The gene set for this investigation was obtained from the TISIDB database, which has a total of 41 chemokines (http://cis.hku.hk/TISIDB/)^23^. The profiles was processed through R software^24^ (version 4.1.1).

### Genetic alteration analysis

In the TCGA-SKCM cohort, GSCA (Gene Set Cancer Analysis)^25^ was carried out to analyze the genetic variation of a total of 41 chemokines, comprising methylation, copy number variation (CNV), and single-nucleotide variation (SNV).

### Consensus clustering for chemokine-related subtypes in SKCM samples

Firstly, gene expression statistics of 470 tumor samples from the TCGA-SKCM dataset and 79 tumor samples from the GSE54467 dataset were combined to create an integrated SKCM cohort. Then, the batch effects of the aforementioned cohorts were eliminated for further analysis using the R packages “limma”^26^ and “sva”^27^ R packages. Based on the chemokine expression profile, we identified two clusters that are related to chemokines using the “ConsensusClusterPlus” R package^28^. To make sure that the classification was stable, there were 1000 repetitions of the algorithm. To investigate how the two chemokine-related clusters function in clinical settings, we then compared the differences between the two subtypes, including gender, age, tumor grade and stage, prognosis, and so on.

### Identification of differentially expressed genes related to chemokines subtypes

Using the R package “limma”^26^, differentially expressed genes (DEGs) between two cancer-related clusters were discovered. The statistical significance was established at |log2FC|≥ 1 and *P* value < 0.05. Then, the “ggplot2”^29^ and “pheatmap” R packages (https://cran.r-project.org/web/packages/pheatmap/index.html) were used to visualize the results.

### Functional and pathway enrichment analysis

To investigate the connection of DEGs to Hallmark pathways, KEGG pathways and Reactome pathways, “GSVA” R package^30^ was used. The MSigDB database provided correlated gene sets (http://software.broadinstitute.org/gsea/msigdb/index.jsp).

### Estimation of the immune cell infiltration

The relationship between risk categorization and immune cell infiltration was under investigation. Three methods were performed as follows: ESTIMATE, CIBERSORT, and ssGSEA with the CIBERSORT algorithm (http://cibersort.stanford.edu/), the ESTIMATE algorithm (https://bioinformatics.mdanderson.org/public-software/estimate/), and the GSVA R package^30^. ESTIMATE algorithm displays the immunological and stromal scores based on RNA-seq statistics from ssGSEA. CIBERSORT algorithm counts the quantity of 22 distinct types of immune cells based on gene expression profiling information. The sum of the 22 immune cell type proportions in each sample was 100 percent. To compare the differences in immunocyte subsets and immunological function between classified clusters, a single-sample gene set enrichment analysis (ssGSEA) was utilized.

### Construction of chemokine-related risk score model based on prognostic DEGs

First, we identified prognostic DEGs using univariate Cox regression analysis. Statistical significance was defined as a *P* value < 0.05. The SKCM cohorts were then divided into different genomic groups based on the predictive DEG expression patterns using consensus clustering analysis. The chemokine-related risk score was calculated applying principal component analysis (PCA). Principal Components (PC) 1 and 2 were decided upon as the feature ratings. SKCM samples were scored using this formula: Risk score = (PC1i + PC2i), where i denotes the expression of each prognostic characteristic gene^31^. According to the appropriate cutoff value, patients were classified into high- and low-risk categories. We examined risk score effects on prognosis using survival and Cox regression analysis on the SKCM dataset.

### The predictive efficiency of chemokine-related risk score model to immunotherapy

Immunophenoscore (IPS) information from the Cancer Immunome Atlas (TCIA) database was used to forecast the immunotherapy sensitivity of SKCM patients (https://tcia.at/)^32^. Higher IPS scores indicate more favorable results following immunocheckpoint inhibitor therapy^33^. To assess immunotherapy options, we compared the IPS values between two risk categories. Furthermore, the applicability of the chemokine-related model was also validated in the external validation set.

### Drug sensitivity analysis based on GDSC

Semi-inhibitory concentration (IC50) values for well-known medicines were generated using the “pRRophetic” R package^34^ in order to examine the clinical utility of chemotherapy regimens. The Wilcoxon signed-rank test was performed to assess IC50 between two different risk groups.

### Statistical analysis

*P* value < 0.05 was regarded statistically significant, if not especially mentioned.

### Ethical approval

The study did not involve human or animal ethics.

## Results

### Genetic and transcriptional alterations of chemokines in SKCM

**Figure 1:**
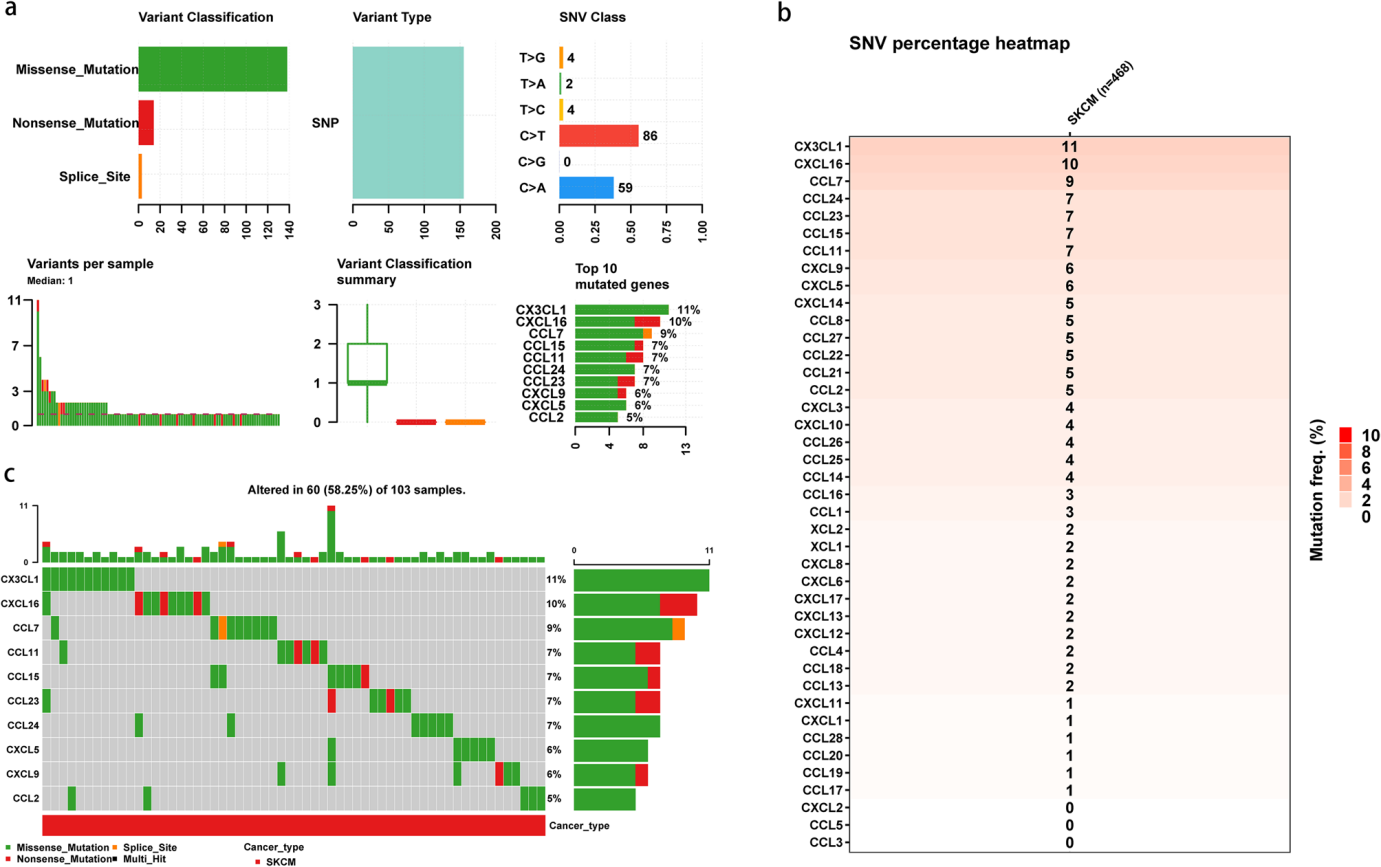
Panoramic view of chemokines mutations in TCGA-SKCM cohort. (**a**) Mutation panorama analysis of chemokines in TCGA-SKCM dataset; (**b**) Summarizes the frequency of deleterious mutations of chemokines in TCGA-SKCM cohort; (**c**) Oncoplot presents the mutation distribution of top 10 mutated chemokines from inputted gene set in sample set of SKCM.

Mutation data from 470 patients was obtained from TCGA-SKCM cohorts to determine the effect of chemokine mutations on patients with SKCM. This study included 41 chemokines in total. Analysis of the somatic mutations pattern of 41 chemokines revealed that SKCM had a relatively high mutation frequency (Fig. 1a). When we examined the overall mutation profile of the TCGA-SKCM patients, we discovered that missense mutations accounted for the vast majority of the mutations. And C > T was the most common type of SNP in SKCM patients. The top ten mutated chemokines were CX3CL1, CXCL16, CCL7, CCL15, CCL11, CCL24, CCL23, CXCL9, CXCL5, and CCL2. The detailed mutation percentage of chemokines in SKCM is shown in Fig. 1b. Oncoplot further presents the mutation distribution and classification of SNV types of top 10 mutated chemokines in TCGA-SKCM (Fig. 1c). CX3CL1 showed the highest mutation frequency (11%), while the second was CXCL16. Three chemokines (CXCL2, CCL5, and CCL3) did not have any mutations.

### Transcriptional variations of chemokines in SKCM

**Figure 2:**
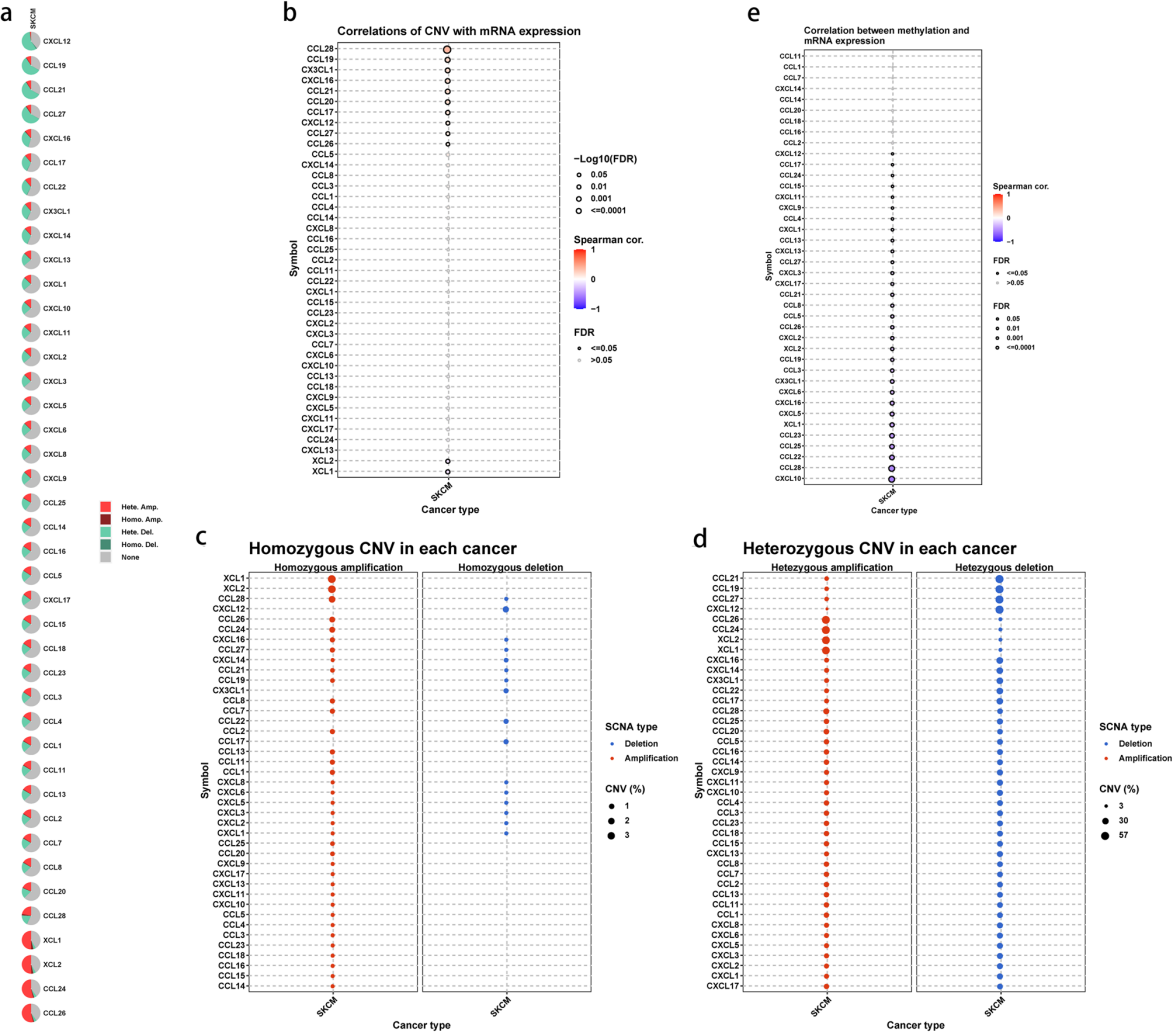
Transcriptional Variations of chemokines in SKCM. (**a**) Pie charts of multiple CNV types for each chemokine in SKCM. (**b**) Correlation of CNVs and chemokine expression. (**c**) The Homozygous CNV pattern of chemokines in SKCM. (**d**) The Heterozygous CNV pattern of chemokines in SKCM. Red represents amplification and blue represents deletion. The size of the dot represents the mutation frequency. (**e**) Correlation between DNA methylation and chemokine expression in SKCM. The color red indicated a positive association, whereas the color blue indicated a negative correlation. The deeper the hue, the higher the correlation index. The size of the bubble represents the FDR.

The pie chart in Fig. 2a provides a summary of Copy Number Variations (CNVs) of chemokines in SKCM. Both heterozygous and homozygous CNVs were observed, including amplifications and deletions. XCL2 and XCL1 exhibited the highest frequency of homozygous and heterozygous amplifications, while CCL21 and CCL19 showed the highest frequency of heterozygous deletions (Fig. 2c,d). Considering that CNVs and DNA methylation can regulate transcript expression^35^, we investigated the relationship between DNA methylation, CNV, and gene expression. As depicted in Fig. 2b, upregulated expressions of 10 chemokines such as CCL28, CCL19, and CX3CL1 were found during CNV formation. A significantly negative correlation was identified between methylation levels and expression levels of most chemokines, particularly for CXCL10 and CCL28 (Fig. 2e).

### Characterization of chemokines in SKCM

**Figure 3:**
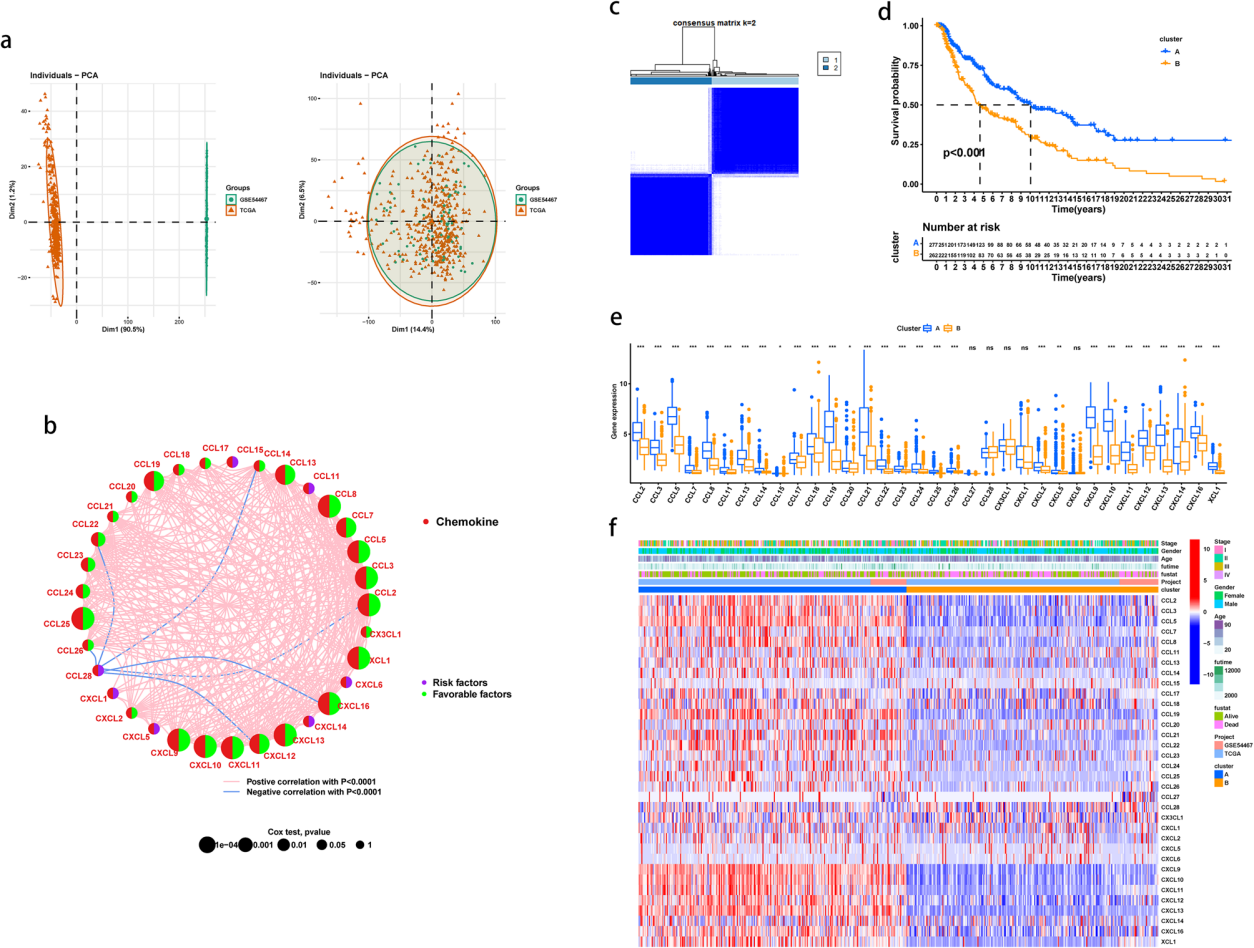
Characterization of chemokines in SKCM. (**a**) Before the removal of batch effects through principal component analysis, the differences between samples obtained from two datasets are illustrated (left); After the removal of batch effects, the differences among samples obtained from two datasets are reduced (Right). (**b**) Prognosis network of interactions between chemokines in SKCM. The P values of each chemokine with respect to the prognosis are shown as circles of different sizes. Purple in the right hemisphere: risk factors for SKCM; green in the right hemisphere: favorable factors for SKCM. Positive or negative correlations of chemokines are linked with lines of different colors (pink: positive, blue: negative, *P* < 0.0001). (**c**) The consensus clustering analysis classified samples into two subgroups when k = 2; (**d**) The difference in survival probability between two clusters; (**e**) The difference in gene expression patterns between two clusters; (**f**) The distinctions in gene expression levels and clinicopathological characteristics. Survival status, genders, ages, project, and cluster group are shown as patient annotations.

To fully understand the expression pattern of chemokines involved in tumorigenesis, two SKCM dataset (470 tumor samples from TCGA-SKCM, and 79 tumor samples from GSE54467) were integrated. It was discovered that batches were greatly decreased after conversion using PCA (Fig. 3a). Total 549 samples and 11,965 genes were obtained. 34 chemokines were used for further analysis. Risk factors, interactions, and regulator relationships of chemokines in SKCM patients were visualized in the prognosis network. Most chemokines were favorable factors for SKCM (Fig. 3b). Using the ConsensusClusterPlus package, we categorized the patients with SKCM based on the expression profiles of the 34 chemokines. Our results showed that k = 2 appeared to be an optimal selection for sorting the entire cohort into cluster A and B (Fig. 3c). Patients in cluster A exhibited a particularly prominent survival advantage compared with cluster B(log-rank test, *p* < 0.001; Fig. 3d), with high-expressed level of most chemokines (Fig. 3e). To further characterize the intrinsic differences between chemokine expression and clinical data among these subtypes, we used a heatmap to depict chemokine-related phenotypes and clinical differences in the TCGA-SKCM and GSE54467 cohorts (Fig. 3f). All of these findings indicated that the patterns of chemokine expression were closely associated to the development and clinical characteristics of SKCM.

### GSVA analysis between two clusters

**Figure 4:**
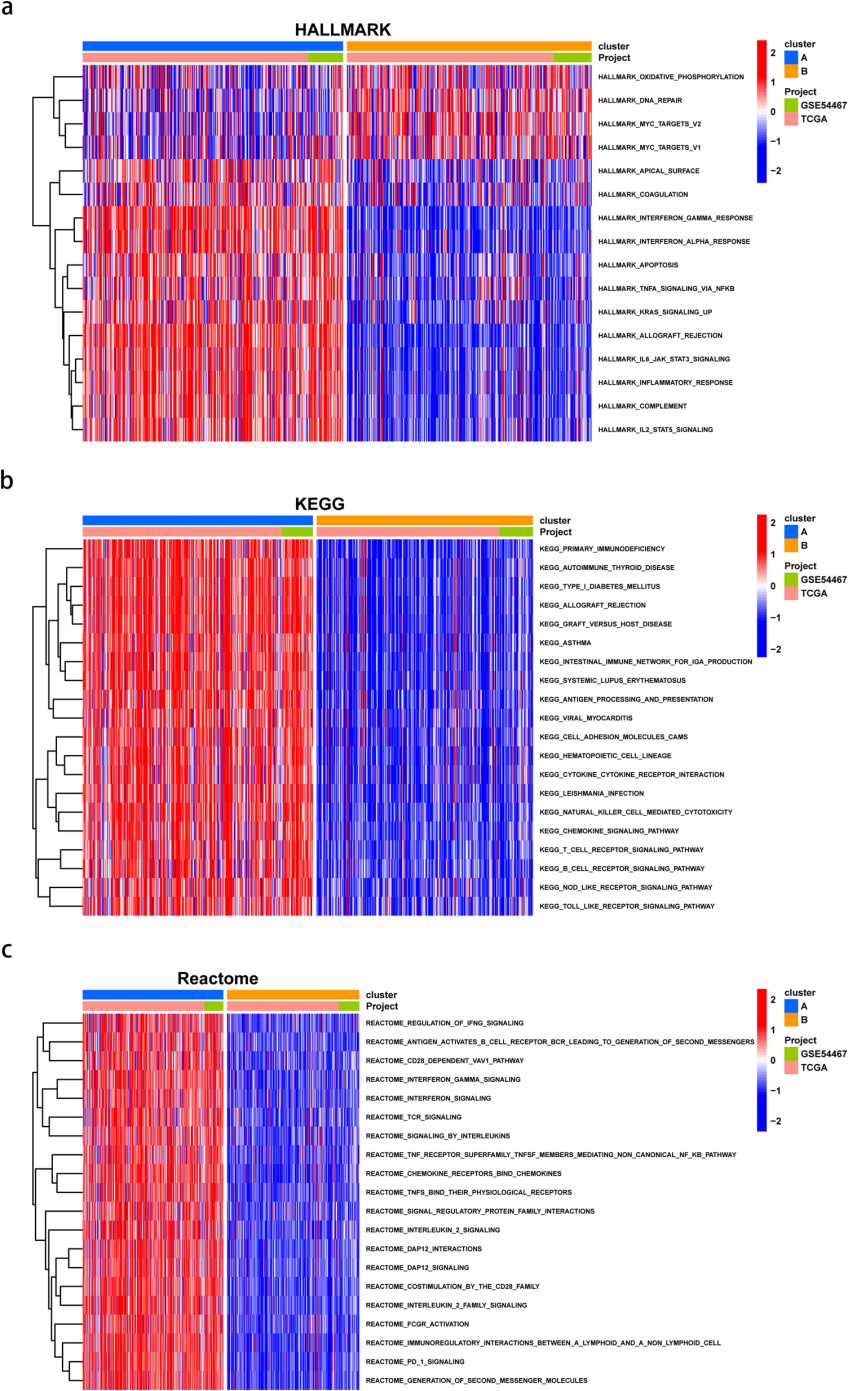
GSVA analysis of chemokines in SKCM. (**a**) HALLMARK pathways between two subtypes. (**b**) KEGG pathways between two subtypes. (**c**) Reactome pathways between two subtypes. The red and blue colors represent pathways that are active and inhibitory, respectively.

GSVA enrichment analysis was conducted to examine functional and biological differences between clusters. HALLMARK analysis showed that cluster B was significantly enriched in oxidative phosphorylation and DNA repair; whereas cluster A was mainly enriched in TGF-β signaling via Nuclear factor-k-gene binding (NF-κB), Apoptosis, inflammatory response, and IL6-JAK-STAT3 signaling (Fig. 4a). KEGG analysis showed that cluster A was enriched in immune-related pathways, such as primary immunodeficiency, B cell and T cell receptor signaling pathway, natural killer cell mediated cytotoxicity. NOD-like and TOLL-like receptor signaling as well as cytokine-cytokine receptor interaction and chemokine signaling pathway were also verified positively related with Cluster A (Fig. 4b). As for Reactome analysis, cluster A was found enriched in PD-1 signaling, TCR signaling, interferon signaling and interleukin-2 family signaling and so on (Fig. 4c).

### The relationship between chemokine expression level and immune cell infiltration in SKCM

**Figure 5:**
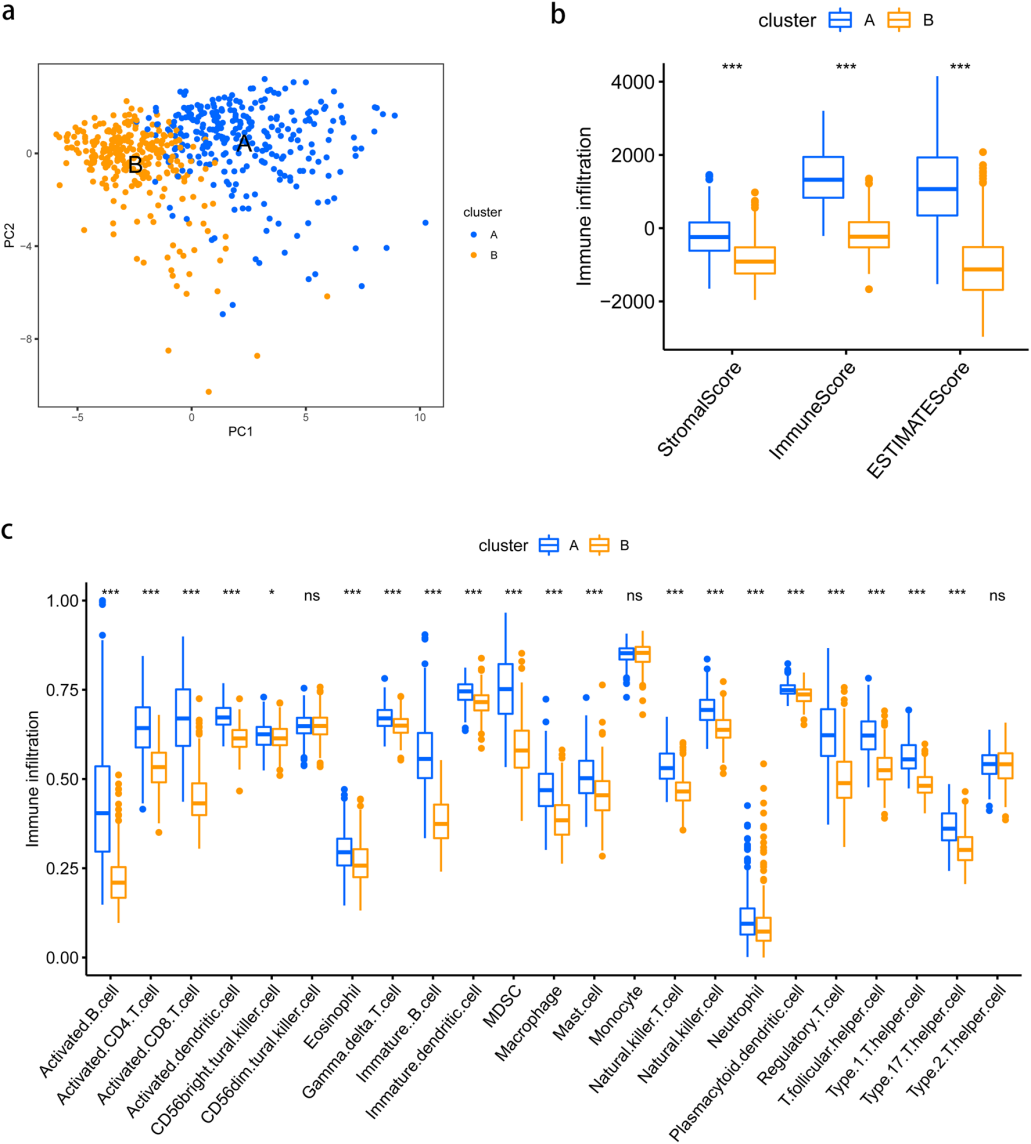
Immune cell infiltration analysis. (**a**) Principal component analysis (PCA) based on chemokines to distinguish two clusters. The PCA distribution of SKCM samples by expression profile of chemokines. Each point represents a single sample; different colors represent the A and B clusters respectively. (**b**) Expression of the TME score in distinct subtypes; (**c**) boxplot represents the 23 immune cell infiltration levels between two chemokines-related subtypes in the TCGA cohort.

We investigated the association between chemokine expression and immune cell infiltration further. Two cluters were clearly differentiated by the principal component analysis (PCA), demonstrating the significant differences between two clusters (Fig. 5a). We evaluated the differences in ImmuneScore, StromalScore, and EstimateScore between two clusters. As shown in Fig. 5b, Cluster B showed a lower ImmuneScore, StromalScore, and EstimateScore than Cluster A, indicating that it had a higher tumor purity than Cluster A. Using the CIBERSORT algorithm, we discovered that, with the exception of CD56dim natural killer cells, all other immune cells in Cluster B were underactivated (Fig. 5c). According to the results indicated above, increased chemokine expression may have caused a greater infiltration of immune cells in SKCM.

### Profiling DEGs between two distinct clusters

**Figure 6:**
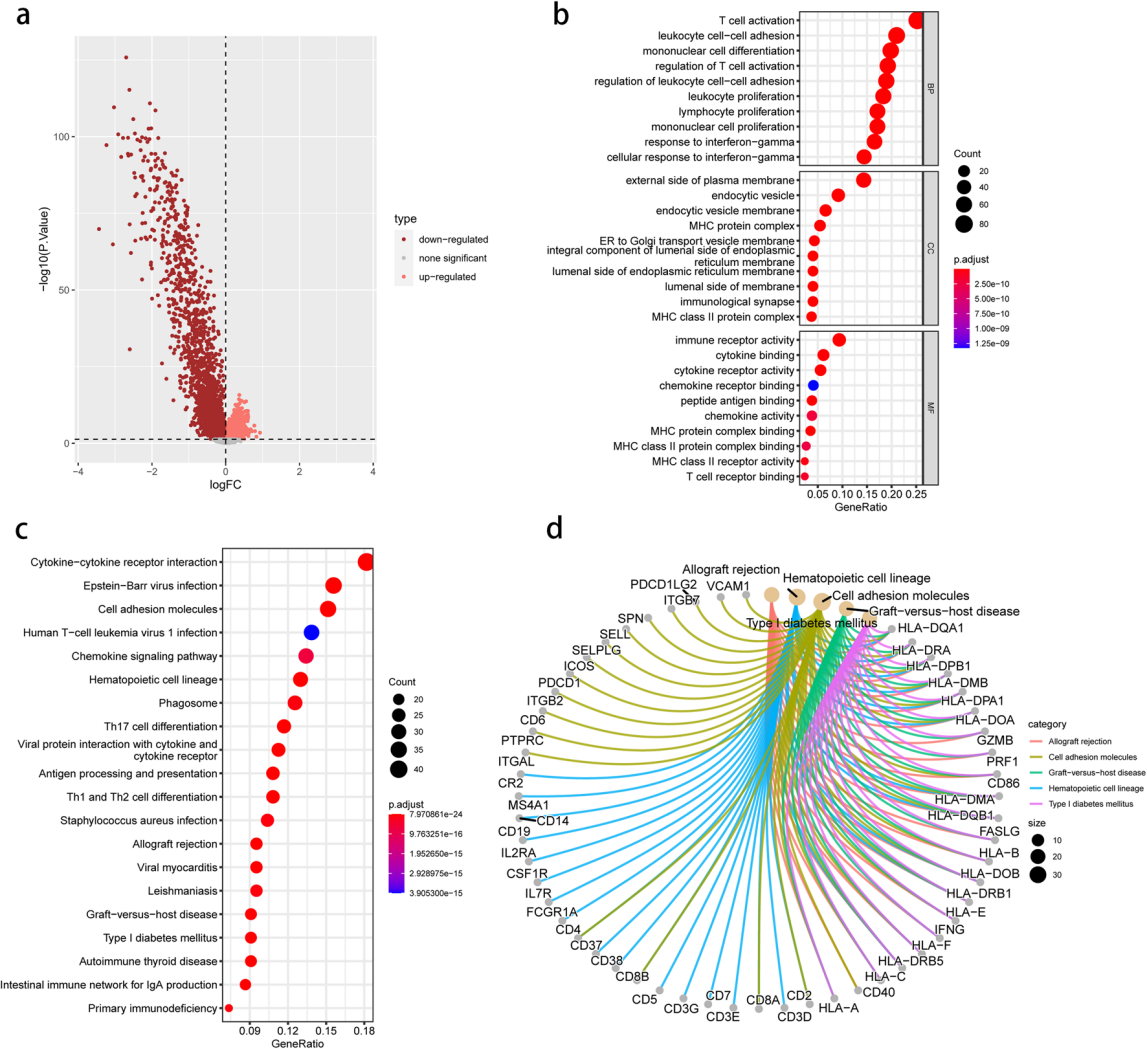
Biological characteristics of chemokine subtype-related DEGs. (**a**) Volcano map showing chemokine subtype-related DEGs. Pink and red colors represent upregulated and downregulated, respectively; (**b**) GO enrichment analysis including biological process (BP), cellular component (CC), and molecular function (MF). (**c**) KEGG enrichment analysis indicating related genes and pathways. (**d**) Circle diagram showing the relationship between DEGs and pathways in KEGG.

Following that, we conducted a DEG analysis between two clusters. Between clusters A and B, 362 DEGs were discovered (Fig. 6a). Furthermore, functional enrichment analysis on those DEGs was done to elucidate the pathways and potential mechanisms of chemokine-related gene clusters. We discovered that DEGs were focused in T cell activation, leukocyte cell–cell adhesion, and T cell activation regulation using GO enrichment analysis (Fig. 6b). Furthermore, the results of KEGG enrichment analysis revealed that DEGs were considerably abundant in cytokine-cytokine receptor interactions, cell adhesion molecules, and the chemokine signaling pathway (Fig. 6c). Figure 6d depicts the connection between genes and pathways in the top 5 KEGG enrichment findings.

### Establishment of chemokines-related gene signature

**Figure 7:**
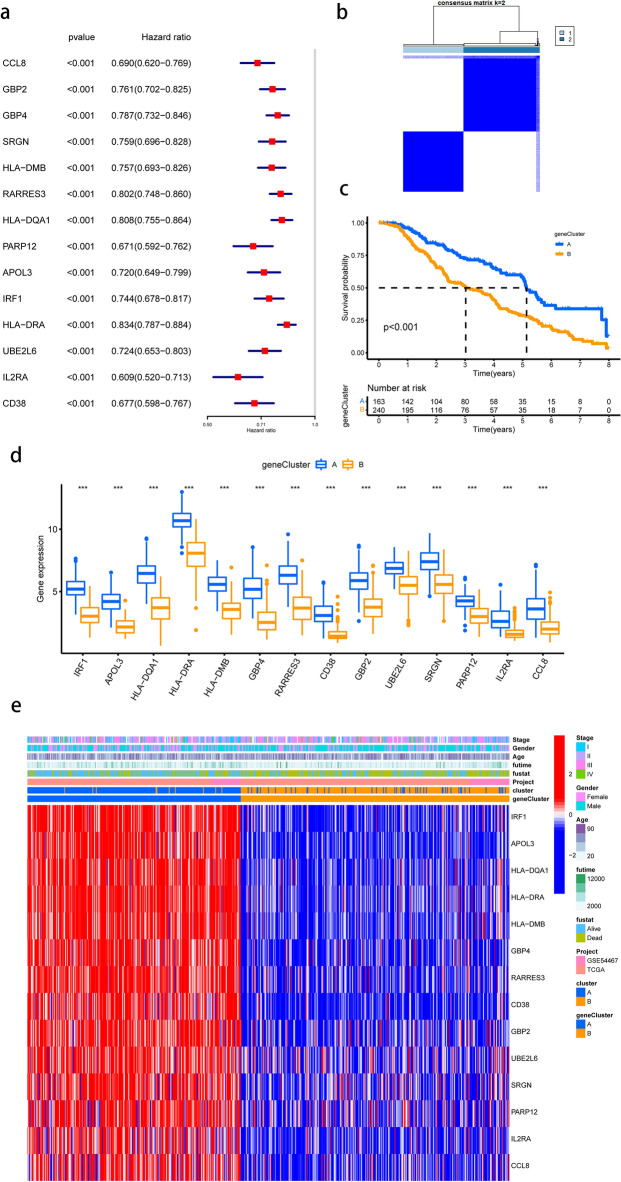
Establishment of chemokines-Related Gene Signature. (**a**) The forest plot of 14 chemokine-associated DEGs with significant prognostic differences using univariate Cox regression analysis. *p < 0.05, ***p < 0.001, ****p < 0.0001, ns: not significant. (**b**) Two distinct clusters based on 14 prognostic DEGs (k = 2, repetition = 5000); (**c**) Overall survival (OS) curve of two clusters; (**d**) The differential expression of chosen DEGs among two gene clusters. (**e**) The heat map was drawn to visualize the expression of prognostic DEGs in distinct chemokine clusters and gene clusters.

The contribution of 362 DEGs on prognosis was studied using univariate Cox regression. 14 genes were rigorously filtered for further investigation, with a p value less than 0.001 defining the significant difference (Fig. 7a). Based on the expression patterns of aforementioned 14 prognostic genes, we obtained two unique gene clusters using consensus clustering analysis (Fig. 7b). Between them, the overall survival curve differed considerably (Fig. 7c). As shown in Fig. 7d, the expression pattern of the aforesaid 14 genes in two clusters was exhibited, demonstrating that 14 genes were all highly expressed in gene cluster A, which caused favorable prognosis. Furthermore, we discovered substantial changes in clinicopathological characteristics across two separate gene groups (Fig. 7e).

### Establishment of chemokine-related prognostic model

**Figure 8:**
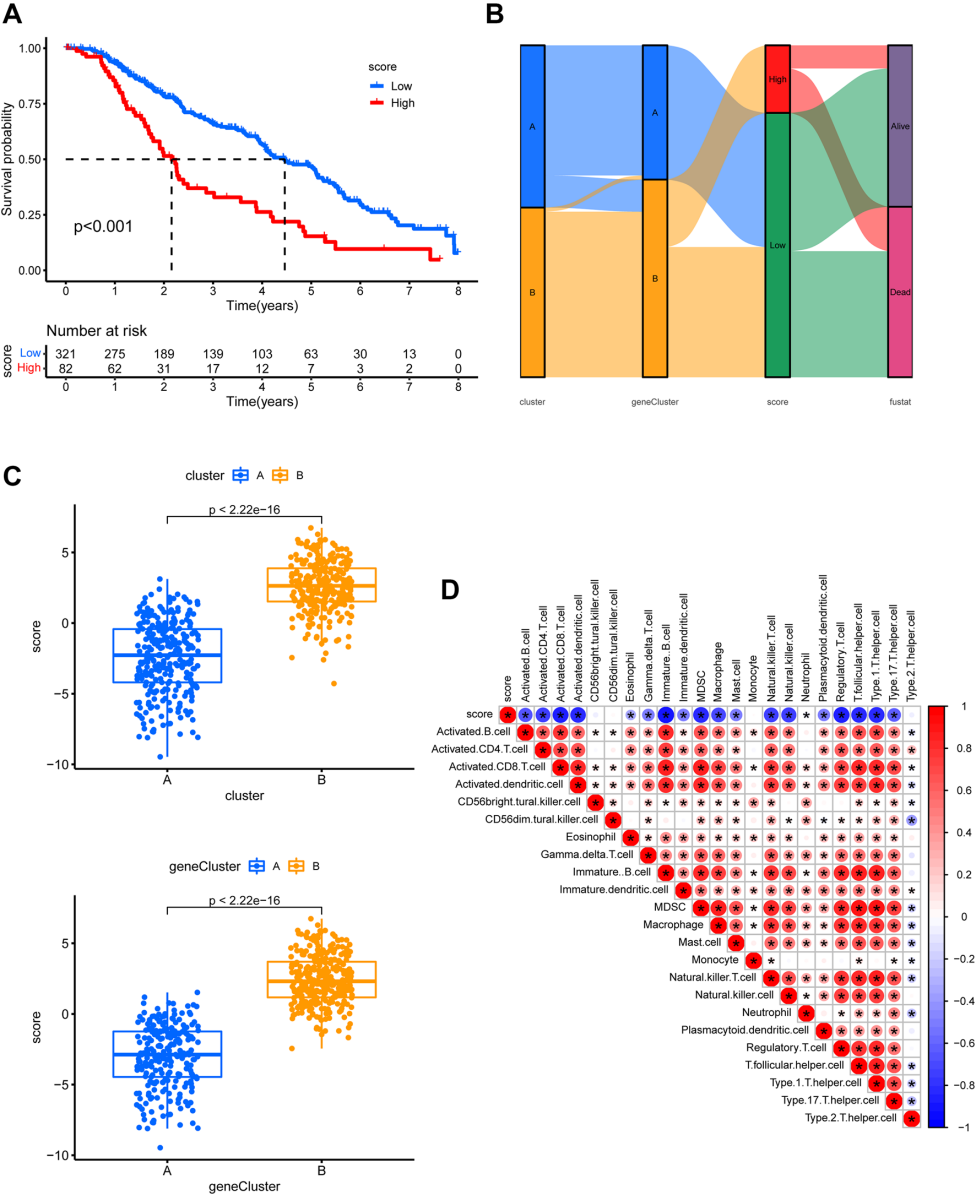
The biological properties of a chemokine-related gene clusters and the establishment of a chemokine-related risk score system. (**A**) Overall survival Kaplan–Meier curves for gene cluster A and B. (**B**) A Sankey diagram depicting the distribution of survival outcomes among different chemokine-related clusters, gene clusters, and risk score groups. (**C**) Chemokine-related risk score differences in SKCM cohorts between two clusters (up) and two gene clusters (down). (**D**) CIBERSORT-based correlation analysis of 22 immune cell types and risk ratings in the TCGA-SKCM dataset. A positive correlation is shown in red, while a negative correlation is shown in blue. The deeper the hue, the higher the correlation index. * denotes *P* < 0.05.

Using PCA, we created a score system based on these prognostic DEGs to assess the chemokine landscape. This score is characterized as the chemokine-related risk score. According to Kaplan–Meier analysis, patients in high-risk group had a shorter life expectancy than those in low-risk group (*p* < 0.001; Fig. 8A). The distribution of survival differences among the different clusters, gene clusters, and two chemokine-related risk score groups was shown using a Sankey diagram (Fig. 8B). We also investigated the relationship between the risk score and chemokine-related clusters as well as gene clusters. Cluster B had a significantly higher risk score than cluster A. The two genetic subgroups yielded similar findings (Fig. 8C). Following that, we looked at the relationship between the risk score and the infiltration level of immune cells. Except for CD56 bright and dim natural killer cells, monocytes, and Type 2 T helper cells, we discovered that risk scores in the SKCM cohort were negatively linked with left types of immune cells out of a total of 22 (Fig. 8D).

### Relationship between the chemokine-related risk score and the response to immunotherapy

**Figure 9:**
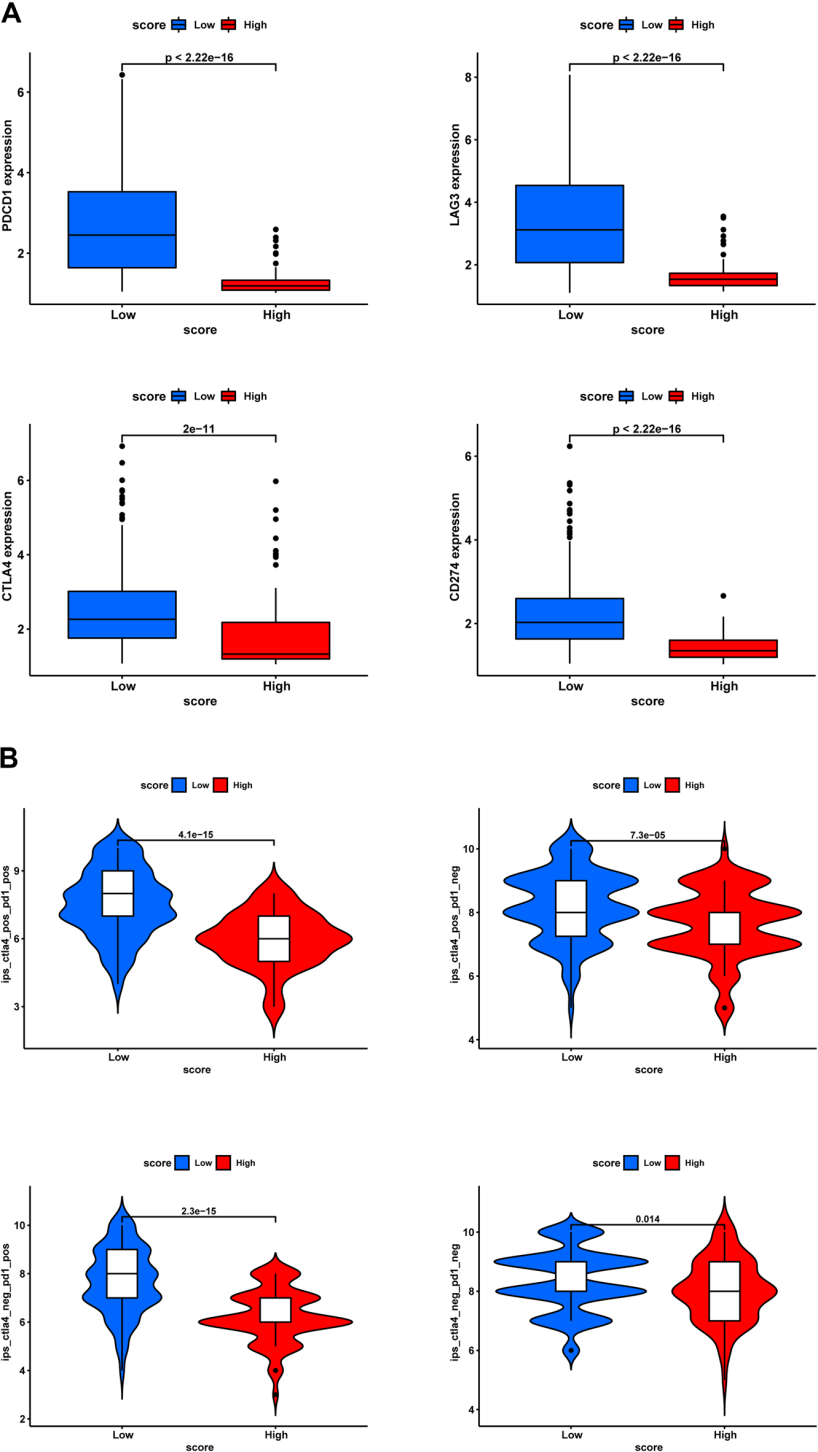
Relationship between the chemokine-related risk score and immunity. (**A**) The degree of immune checkpoint expression in SKCM patients. The low-riskscore group had greater levels of all four checkpoints than the high-riskscore group. (**B**) The association of IPS and risk score categories in SKCM patients. The low-riskscore group had greater levels of IPS-CTLA4-/PD-L1-, IPS-CTLA4-/PD-L1+, IPS-CTLA4+/PD-L1-, and IPS-CTLA4+/PD-L1+ (all *p* < 0.05).

Due to immunotherapy using immune checkpoint inhibitors (ICIs), the prognosis for cancer patients has considerably improved. A recent study demonstrated that IPS based on immunogenicity can help predict the effectiveness of immunotherapy. The link between the risk score and IPS in SKCM was then examined to determine whether it might be used to predict the outcomes of immunotherapy. Differential expression of immunological checkpoints was lower in the high-risk group (Fig. 9A), implying that immunotherapy was less effective in this group. Moreover, IPS-CTLA4-/PD-L1-, IPS-CTLA4-/PD-L1+, IPS-CTLA4+/PD-L1-, and IPS-CTLA4+/PD-L1+ were substantially greater in the low-risk group (Fig. 9B), suggesting that immunotherapy would be more effective for individuals with low risk scores.

### Validation of chemokine-related risk score model in two independent external cohorts

**Figure 10:**
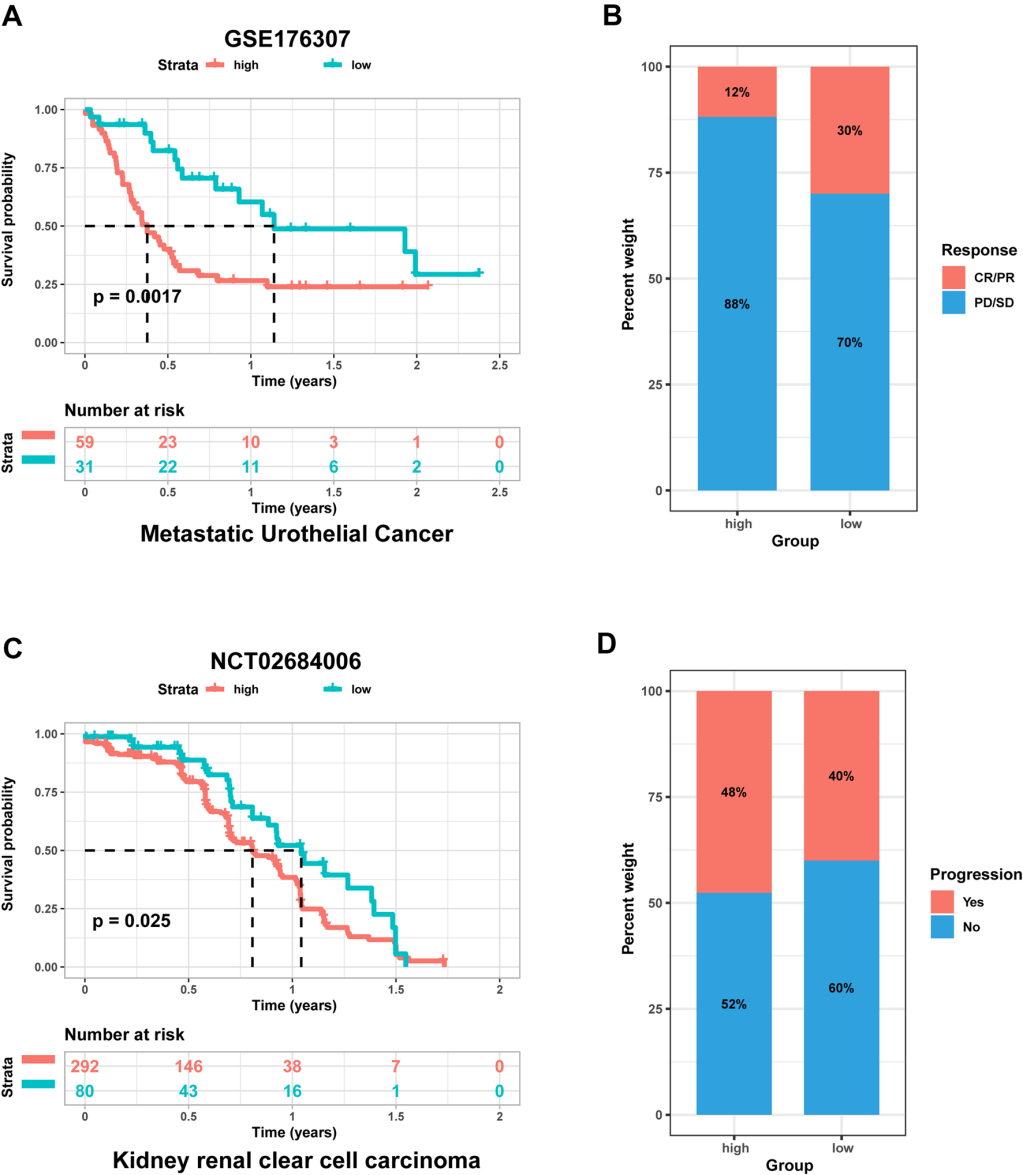
Validation of chemokine-related risk score model in two independent external cohorts. (**A**) and (**C**): Kaplan–Meier curves in two test cohorts based on chemokine-related risk score model; (**B**) and (**D**): Predictive effectiveness of chemokine-related risk score model to immunotherapy in two test cohorts.

The test sets were selected from two datasets: the KIRC dataset (NCT02684006)^36^, which included samples treated with anti-PD-1 therapy, and the metastatic urothelial cancer dataset (GSE176307)^37^, which included samples treated with anti-PD-1 or anti-PD-L1 therapy. The aforementioned algorithm was used to calculate the risk scores for each patient in the testing sets. Patients were then split into high-risk and low-risk groups based on the median risk value. Remarkably, compared to the low-risk score patients, Kaplan–Meier curves revealed that high-risk score patients had worse prognosis in GSE176370 as well as NCT02684006 (Fig. 10A,C). Additionally, the chi-squared test showed that patients with low risk levels had higher rates of CR/PR but lower rates of SD/PD when compared to those with high risk scores (Fig. 10B). Patients with CR and PR showed significantly longer survival times than those with PD and SD, according to several therapeutic impact evaluation indices. In a similar vein, the high-risk-score patients in another test cohort displayed a higher rate of advancement, demonstrating that this risk score model was very effective at predicting prognosis (Fig. 10D).

### Potential role of risk score model in chemotherapeutic value

**Figure 11:**
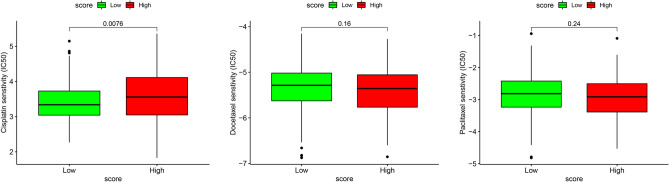
Drug sensitivity analysis. The IC50 values of three chemo drugs in the low- and high-risk groups.

Given that immune/chemotherapy is one of the most often used treatments for SKCM, we employed the pRRophetic methodology to assess drug efficacy. We calculated the IC50 for each sample in the TCGA-SKCM cohort based on the prediction model. Due to the fact that cisplatin, docetaxel, and paclitaxel were common cytotoxic drugs in melanoma, we presented relevant results in Fig. 11. Significant variations in IC50 estimations were found between the high- and low-risk groups, suggesting that the low-risk group was more susceptible to cisplatin (Figs. 11). These results implied that this chemokine-related score model might also have potential predictive value on chemotherapeutic efficacy of Cisplatin in SKCM.

## Discussion

The TME plays a critical role in the anti-tumor response^8^. Chemokines and their receptors may shape the TME and then affect the proliferation, invasion and metastasis of tumor cells^38,39^. To identify their functions in SCKM, we first systematically analyzed the mutation frequency and CNV alteration of chemokines by using the information from TCGA database. As reported, gene mutation plays a pivotal role in melanoma^40,41^. We found that the expression of most chemokines was dysregulated in SKCM. Among them, CX3CL1 is the most prevalent and has recently been discovered as a pro-angiogenic factor in myeloma patients^42^. It has been reported that CX3CL1 plays a crucial role in regulating cell adhesion, migration, and survival of human cancer cells^43–45^. ICAM-1 also plays a key role in cell adhesion to endothelium and tumor development, as well as cell proliferation, invasion, and angiogenesis. The CX3CL1-CX3CR1 axis was found to be essential for promoting ICAM-1 regulation of tumor metastasis^46^. Furthermore, highly expressed and active ICAM-1 promoter responsive genes were identified in melanoma cells^47^. Additionally, CX3CL1 were reported to be overexpressed in melanoma cells and tissues^48^. In the present study, CX3CL1 did not exhibit differential expression between the two clusters. Therefore, we hypothesize that mutations in CX3CL1 may also impact the function of ICAM-1, thereby influencing melanoma cell proliferation, invasion, and angiogenesis; however further in vitro experiments are required.

Next, we constructed two clusters of SKCM patients based on 41 chemokines for further investigation. By comparing with patients in cluster B, patients in cluster A had better OS accompanied by high expression of most chemokines. As expected, cluster A was significantly enriched in B cell and T cell receptor signaling pathway (KEGG), TNF signaling via NF kappa-B (HALLMAPK). In most normal cells, NF-κB exists in its inactive form, but constitutive activation of NF-κB has been noted in almost all cancers. Among all growth factors, TNF-α is one of the most potent activators of NF-κB^49^. This circumstance also exists in melanoma^50^. NF-κB–mediated activation in the immune system has the potential to suppress tumor growth, in part through the production of growth inhibitory cytokines. Activation of NF-κB is usually characterized by production of cytokines and chemokines^51^. Therefore, we postulate that increased chemokine expression in SKCM can coincide with NF-κB activation, trigger immune cell infiltration, and act as a preventative measure against melanoma.

As reported, chemokines are essential for the direct migration of immune cells into the tumor microenvironment to generate an effective anti-tumor immune response. However, studies have shown that immune cells in the tumor microenvironment may exert effective anti-tumorigenic or pro-tumor responses, and the balance between these responses depends on the stage of tumorigenesis, the state of immune cell activation, and the expression of chemokines to effector cells and regulatory target cells^52,53^. In this study, two subtypes based on chemokines expression profile were also characterized by a significant immune infiltration. Our results indicated that the infiltration of CD4 T cells, CD8 T cells, neutrophils, macrophages and dendritic cells was positively associated with the chemokine expressions, suggesting that high expression level of these chemokines leading to high immune cells infiltrations and a better prognostic factor. Similarly, high expression of 32 chemokines and receptors, namely CCL2,4-5,7-8,13,22-25, CCR1-9, CXCL9-13,16, CXCR3,5,6, XCL1-2, and XCR1, in reginal lymph node tissue in the SKCM patients were found associated with good outcomes in previous study^54^.

In order to better cluster the tumors and construct a good prognostic risk model, we firstly obtained 14 prognosis-related DEGs between aforementioned two clusters using Cox regression analysis (CCL8, GBP2, GBP4, SRNG, HLA-DMB, RARRES3, HLA-DQA1, PARP12, APOL3, IRF1, HLA-DRA, UBE2L6, IL2RA and CD38). We then used prognosis-related DEGs to cluster SKCM data and found that SKCM could be effectively divided into 2 gene clusters, and the prognosis of the gene cluster A and B was significantly different. Furthermore, a prognostic risk model was constructed based on the expression pattern of aforementioned prognosis-related DEGs. Our analysis found a positive correlation between the risk score and OS, but a negative correlation between the risk score and immune cell infiltration, suggesting that the risk score was an independent prognostic predictor for SKCM patients.

The IPS can predict response to immunotherapy with CTLA-4 and PD-1 blockers^55^. Our analysis showed that patients in low-risk group had a higher IPS, indicating that they were more sensitive to treatment against PD1 or CTLA4. This may also be related to the fact that there is more infiltration of immune cells but greater expression of immune-inhibitors such as PD1 and CTLA4. Two more independent cohorts (GSE176307: anti-PD-1, and anti-PD-L1; NCT02684006: anti-PD-1, and IMvigor210: anti-PD-L1) were analyzed, and the results showed that patients in the low-risk score category were more responsive to anti-PD-1/PD-L1 medication and had longer survival times. This offers more convincing proof. Contingent upon the degree of chemokine articulation, the composition of tumor-infiltrating immune cells might change and in the long run affect the immune response to tumor regression^12,20^. CXCL10 has been shown to be a crucial chemokine for drawing CD8+ T lymphocytes into melanoma tumors in preclinical models. Immune checkpoint inhibitory response is closely correlated with CXCL10 expression, which can predict response without an immune cell invasion^56^. It may be possible to consider methods to increase the production of chemokines such as CXCL10 in the TME in order to increase the effectiveness of checkpoint blockade. Chemokines secretion (CXCR6, CXCL9, CCL5, and CCR5) was also shown to predict response to anti–PD-1–directed therapy in melanoma patients^57^. In addition to immunotherapy prediction, we also predicted the efficacy of chemotherapy (cisplatin, docetaxel, and paclitaxel) in the high- and low-risk groups. And we observed significant differences in IC50 estimates between the high- and low-risk groups of Cisplatin. The high-risk group had higher IC50 values, indicating that these patients were less sensitive to Cisplatin. These findings imply that the chemokine-based risk score model is not only beneficial for predicting prognosis, but also for the treatment efficacy of SKCM patients. However, it should be mentioned that our results need additional in vitro or in vivo validation.

To conclude, we developed a new SKCM classification system and a risk score model on the basis of the chemokine-related subtype of the tumor. As a prognostic model in melanoma patients, this risk score model also produced meaningful results in predicting prognosis and treatment efficiency.

## Data Availability

The datasets generated and analysed during the current study are available in the Gene Expression Omnibus database (http://www.ncbi.nlm.nih.gov/geo) and UCSC-XENA database (https://xenabrowser.net/datapages/).
